# Glycerol Monolaurate (GML) inhibits human T cell signaling and function by disrupting lipid dynamics

**DOI:** 10.1038/srep30225

**Published:** 2016-07-26

**Authors:** Michael S. Zhang, Aline Sandouk, Jon C. D. Houtman

**Affiliations:** 1 Department of Microbiology, Carver College of Medicine, University of Iowa, Iowa City, Iowa 52242, United States.

## Abstract

Glycerol Monolaurate (GML) is a naturally occurring fatty acid widely utilized in food, cosmetics, and homeopathic supplements. GML is a potent antimicrobial agent that targets a range of bacteria, fungi, and enveloped viruses but select findings suggest that GML also has immunomodulatory functions. In this study, we have mechanistically examined if GML affects the signaling and functional output of human primary T cells. We found that GML potently altered order and disorder dynamics in the plasma membrane that resulted in reduced formation of LAT, PLC-γ, and AKT microclusters. Altered membrane events induced selective inhibition of TCR-induced phosphorylation of regulatory P85 subunit of PI3K and AKT as well as abrogated calcium influx. Ultimately, GML treatment potently reduced TCR-induced production of IL-2, IFN-γ, TNF-α, and IL-10. Our data reveal that the widely used anti-microbial agent GML also alters the lipid dynamics of human T cells, leading to their defective signaling and function.

Glycerol Monolaurate (GML) is a naturally occurring fatty acid molecule and antimicrobial agent^1^. GML suppresses the growth and virulence of numerous gram positive and gram negative bacteria, fungi, and enveloped viruses^2^,^3^,^4^,^5^,^6^. GML is on the FDA’s Generally Recognized as Safe list (GRAS)^7^ and is incorporated in various products such as deodorants, lotions, and cosmetics. It is also widely available as a homeopathic supplement, and is extensively used as a food preservative and emulsifier^8^,^9^. Topical GML is being tested as a treatment and/or preventative measure for Toxic Shock Syndrome, HIV transmission, and surgical site infections^10^,^11^,^12^. Thus, GML is an effective antimicrobial that comes in regular contact with the general public through its extensive commercial and therapeutic uses.

Intriguingly, several studies have suggested that GML suppresses inflammatory processes, indicating that GML may also have an immunomodulatory role. GML treated T cells have decreased cellular proliferation when stimulated with phorbol 12-myristate 12-acetate (PMA), concanavalin A (ConA), and Toxic Shock Syndrome Toxin-1 (TSST-1)^13^. Additionally, GML treatment decreases inositol triphosphate (IP_3_) generation upon TSST-1 stimulation, indicating decreased phospholipase Cγ1 (PLC-γ1) enzymatic activity^14^. These studies suggest that GML potentially alters T cell activation. However, how GML treatment affects the full T cell activation response from signaling events to effector functions is not well understood. In addition, the mechanism behind how GML mediates suppressed T cell activation is completely unknown.

To directly address these questions, we mechanistically characterized how GML affects human primary T cell activation. We observed that GML treatment drastically altered the balance of ordered vs. disordered lipid phases in the membrane. As a consequence, TCR-induced LAT, PLC-γ, and AKT microcluster formation from aggregation of smaller nanocluster units, PI3K-AKT signaling axis, and calcium influx were potently inhibited by GML treatment. Overall these defects resulted in decreased TCR induced cytokine production. This is the first mechanistic evidence showing that GML suppresses the human immune system. These findings further the current understanding of this compound but also open up the possibility that GML could serve as a potent immunosuppressant.

## Results

### GML treatment suppresses TCR and CD28 induced cytokine production without affecting viability

**Figure 1:**
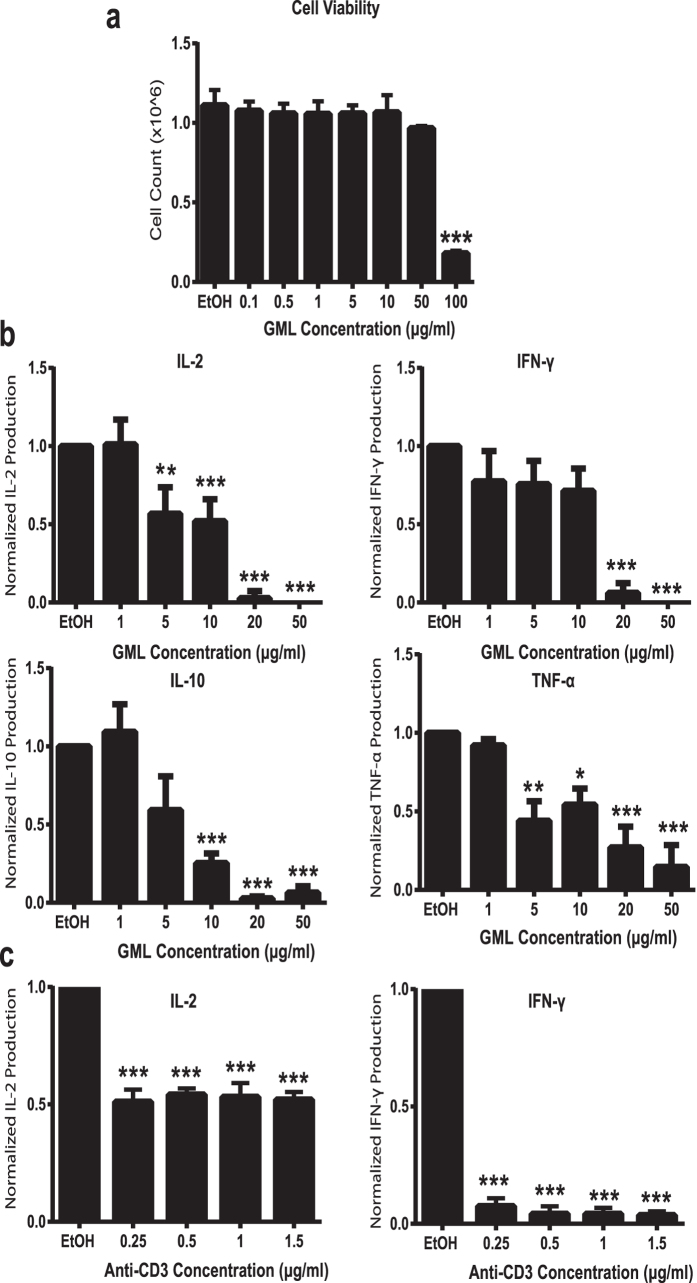
GML suppresses T cell effector functions without affecting cell viability. (**a**) APBTs were treated with varying doses of GML or 0.5% ethanol for 24 hours. Cells were then labeled with Tryphan blue dye and viable cells were counted under light microscopy. Data is shown from 3 independent experiments. (**b**) Human APBTs were treated with 0.5% ethanol or varying doses of GML and stimulated for 24 hours. Extracellular cytokine levels were measured by ELISA for IL-2 (Upper Left), IFN-γ (Upper Right), IL-10 (Lower Left), and TNF-α (Lower Right). Cytokine levels were normalized to ethanol control and data were compiled from at least 3 independent experiments. (**c**) APBTs were treated with 10 μg/ml of GML for IL-2 ELISA (Left), 20 μg/ml of GML for IFN-γ (Right), or 0.2% ethanol control. Extracellular cytokine levels were measured by ELISA. Cytokine levels were normalized to ethanol control and data were compiled from 3 independent experiments. ** denotes p < 0.01 and *** denotes p < 0.001 in one way ANOVA statistical analysis.

Previous studies suggested that GML modulates human T cell function^13^,^14^. However, no studies have comprehensively examined the effects of TCR-mediated T cell activation or identified potential mechanisms for the effects of GML on TCR-mediated T cell activation. To address these knowledge gaps, we treated human activated peripheral blood T cells (APBTs) with solubilized GML or ethanol vehicle control. We first examined whether GML treatment affected T cell viability. APBTs were treated with varying doses of GML for 24 hours and live cells were counted using tryphan blue staining. We observed that GML doses between 0.1 μg/ml and 50 μg/ml caused little to no change in cell viability, while doses above 50 μg/ml drastically decreased viability (Fig. 1a). Next, we measured how GML affects the TCR-induced production of cytokines using ELISA. We found that GML significantly suppressed the production of IL-2, IFN-γ, TNF-α, and IL-10 in a dose dependent manner. (Fig. 1b) We also observed that GML potently suppressed IL-2 and IFN-γ release at anti-CD3 doses ranging from 0.25 μg/ml to 1.5 μg/ml for TCR stimulation (Fig. 1c). Due to the highly variable nature of data from human donors, cytokine production values were normalized to ethanol control groups. Scatter plots of non-normalized data from individual human donors are shown in Supplementary Fig. S1.

### GML selectively inhibits cytosolic calcium influx and PI3K-AKT signaling axis without affecting other components of TCR and CD4 induced signaling events

Upon TCR activation, the tyrosine kinase ZAP-70 is phosphorylated, resulting in increased activity for its substrate adaptor proteins SLP-76 and LAT. The phosphorylation of SLP-76 and LAT then drives the phosphorylation/activation of PLC-γ1, a critical enzyme responsible for cleaving PIP_2_ into IP_3_, the second messenger responsible for calcium influx^15^,^16^,^17^. Additionally, phosphorylated SLP-76 regulates the recruitment and activation of the regulatory p85 subunit of PI3K^18^. This association in combination with phosphorylated LAT drives PI3K activation of AKT^19^. Finally phosphorylated SLP-76 and LAT drive the activation of other downstream signaling proteins including p38 and ERK kinases^15^. In T cells stimulated with TSST-1, GML treatment has been observed to decrease IP_3_ levels^14^, suggesting that GML suppresses T cell functions by interfering with components of the T cell signaling cascade.

**Figure 2:**
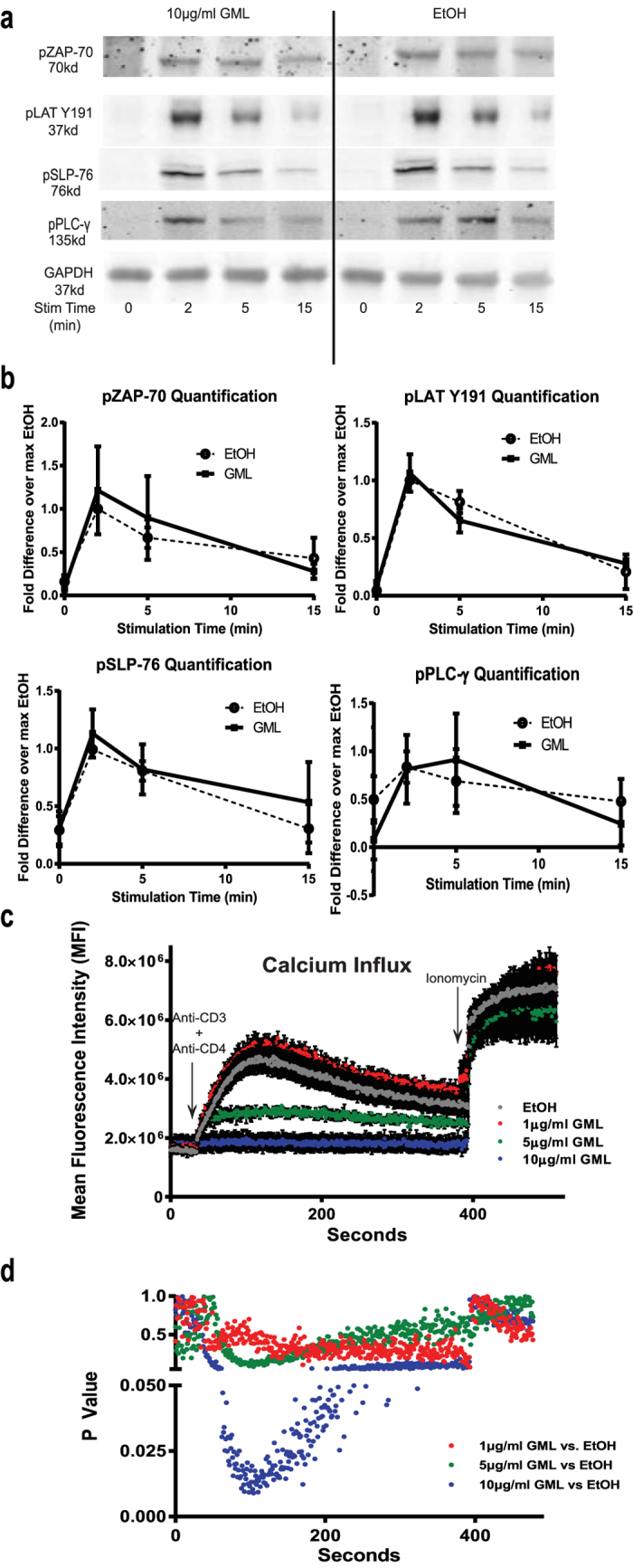
GML inhibits SLP-76 phosphorylation and cytosolic calcium influx. (**a**) APBTs were treated with 10 μg/ml of GML or 0.1% ethanol and stimulated for various times. Phosphorylation of ZAP-70 Y493, LAT Y191, SLP-76 Y128, and PLC-γ Y783 were assessed by immunoblotting. (**b**) Quantification of Western blots shown in (**a**) from 5 independent experiments with different human donors are shown. *** denotes p < 0.001 in Student’s t test. (**c**) APBTs were treated with 0.2% ethanol or varying doses of GML, stimulated, and calcium influx curve was measured in real time for 6 minutes by flow cytometry. 2 μg/ml of Ionomycin was then added to control for intracellular calcium. Data shown is compiled from 3 independent experiments. (**d**) Mean fluorescence intensities of calcium influx curves for APBTs treated with varying doses of GML underwent Student’s t test analysis against ethanol vehicle control. P values from Student’s t test are shown.

**Figure 3:**
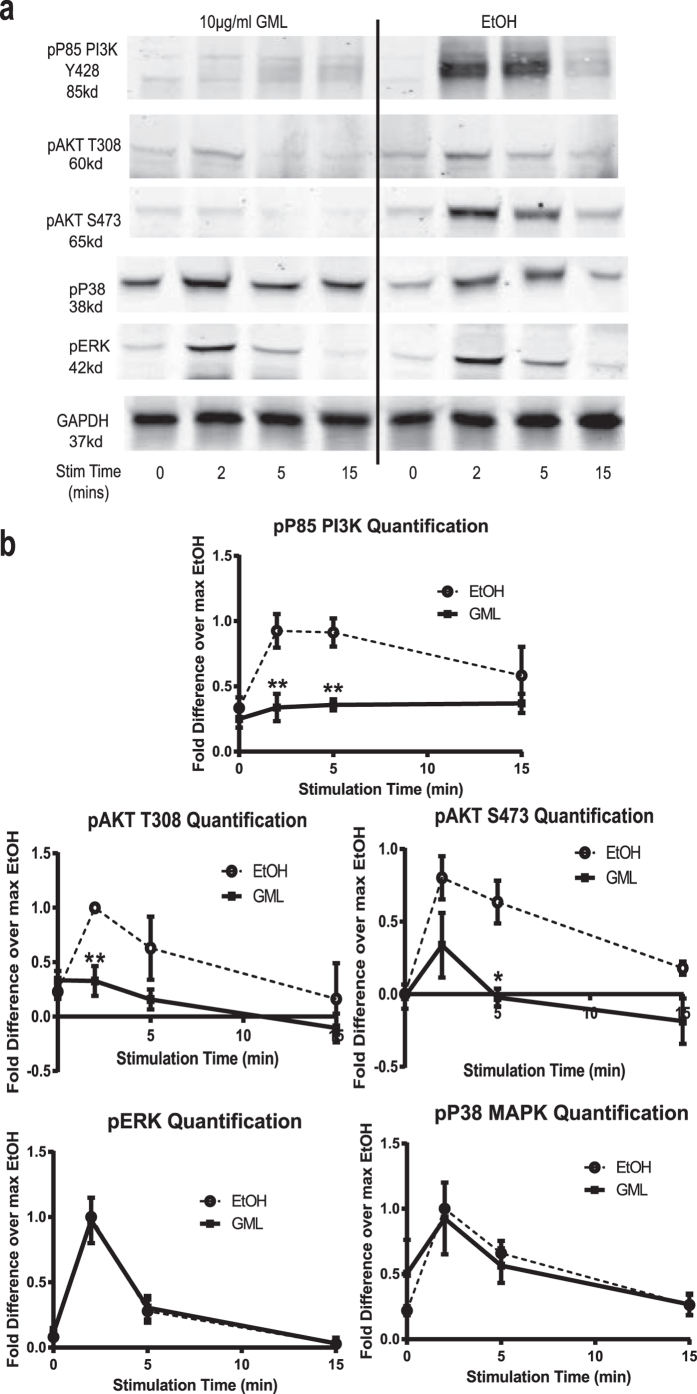
GML interferes with AKT but not MAPK signaling. (**a**) APBTs were treated with 10 μg/ml of GML or 0.1% ethanol and stimulated for various times. Phosphorylated p85 regulatory subunit of PI3K Y458, Akt T308 and S473, p38 MAPK T180/Y182, and ERK1/2 T185/Y187 were assessed by immunoblotting. (**b**) Quantification of immunoblots shown in (**a**) from 5 independent experiments with different human donors are shown. * denotes p < 0.05, and ** denotes p < 0.01 in Student’s t test.

To address if GML similarly affected TCR-mediated signaling, we measured the phosphorylation and activation of various signaling proteins in TCR-activated APBTs in the presence or absence of GML. GML treatment had no effect on site specific phosphorylation of SLP-76 (Y128), ZAP-70 (pY319), LAT (pY191), or PLC-γ1 (Y783) (Fig. 2a,b). However recent studies have shown that phosphorylation of PLC-γ1 on tyrosine 783 does not guarantee enzymatic activity^20^,^21^. To test if GML alters the functional outputs of PLC-γ1 activation, we examined the effects of GML on calcium influx in APBTs using a calcium sensitive dye, Fluo-4M. Interestingly, increasing doses of GML significantly suppressed calcium influx in TCR-induced T cells with complete abrogation of calcium influx at the 10 μg/ml dose of GML (Fig. 2c,d). Downstream, GML treatment significantly reduced the phosphorylation of the regulatory p85 subunit of PI3K. Phosphorylation of both T308, the directly target of PI3K kinase activity, and S473 residues of AKT were also suppressed. However, GML treatment had minimal effects on the phosphorylation of MAPKs ERK1/2 and p38 (Fig. 3). These data show that GML does not result in global suppression of TCR-induced signaling but instead causes a targeted signaling defect in calcium signaling and the PI3K-AKT axis.

### GML prevents TCR induced formation of phosphorylated LAT but not SLP-76 nucleated signaling microclusters at the plasma membrane

**Figure 4:**
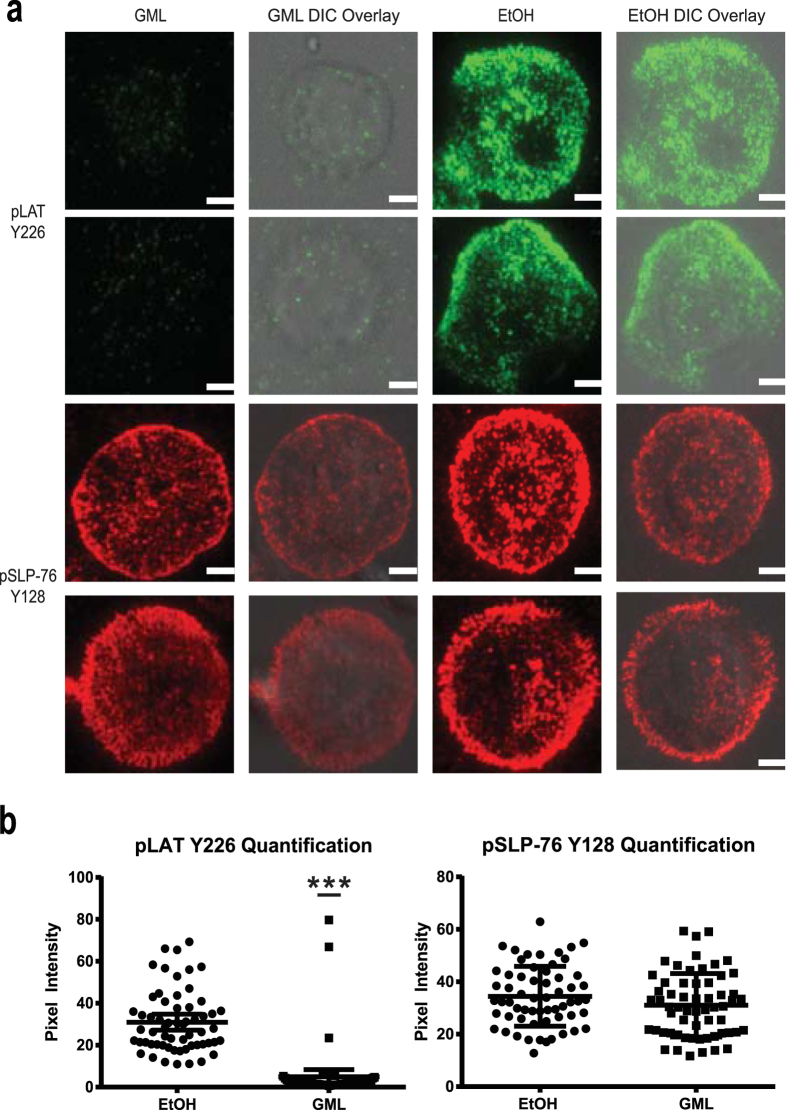
GML prevents membrane clustering of phosphorylated LAT but not SLP-76. (**a**) APBTs treated with 10 μg/ml of GML or 0.1% ethanol were stimulated in glass covered chamber slides. They were then fixed, permeabilized, and stained with antibody specific for phosphorylated LAT Y226 and SLP-76 Y128. Cells were imaged using TIRF microscopy. Individual cells are shown. White bar scale indicates 4 μm in length. (**b**) Pixel intensities of images obtained in (**a**) were quantified using ImageJ. Pixel intensities on the median axis of each cell were quantified and averaged. Scatter plot distributions with 95% confidence intervals of 60 cells from 2 independent experiments are shown. *** denotes p < 0.001 in Student’s t test.

TCR activation induced signaling cascades occur in membrane localized microclusters that are highly enriched in signaling molecules to allow rapid signal propagation to downstream effectors^22^,^23^,^24^. The formation of these microclusters that are visible under fluorescent microscopy is highly dependent on TCR activation induced aggregation of smaller nanocluster units of LAT and its signaling partners (<5 LAT molecules) that are not visible under standard fluorescent microscopy^25^,^26^,^27^. Additionally, nanoclusters of SLP-76 combine into microclusters at the periphery of LAT clusters upon TCR activation and this phenomenon is separable from LAT microcluster formation^27^. T cells lacking the adaptor protein GRB2 have severely reduced LAT nucleated microcluster formation, resulting in suppressed calcium influx in the presence of detectable phosphorylation of PLC-γ1 Y783^21^. This signaling phenotype is intriguingly similar to the outcome of GML treatment. Therefore it is plausible that GML may alter LAT and/or SLP-76 clustering to inhibit PLC-γ1 enzymatic function and intracellular calcium influx. To examine if GML affects LAT and/or SLP-76 nucleated microclustering at the plasma membrane, we stimulated control and GML-treated cells with plate-bound anti-CD3 and then visualized these clusters using total internal reflection fluorescence (TIRF) microscopy by staining with appropriate antibodies. Ethanol treated controls developed extensive membrane microcluster aggregation of both upstream adaptor proteins, phosphorylated LAT and SLP-76. In contrast, T cells treated with GML exhibited drastically less clustering of phosphorylated LAT but surprisingly had no effect on phosphorylated SLP-76 microclusters (Fig. 4a). Quantification of the pixel intensity in the central axis of individual cells showed that GML treated cells had significantly reduced LAT but not SLP-76 microcluster pixel intensity compared to ethanol control (Fig. 4b). Interestingly, similar to GML’s effects on total LAT phosphorylation, GML does not alter LAT phosphorylation in the membrane fraction (Supplemental Fig. S2). While GML’s effects on the clustering of LAT does not fully correlate with its effects on LAT phosphorylation, multiple reports show that detection of protein phosphorylation levels by immunoblotting do not always directly correlate with membrane localized clustering measured by TIRF microscopy^21^,^28^. This result shows that GML selectively suppresses the membrane-localized formation of LAT but not SLP-76 microclusters.

### GML prevents the aggregation of AKT and phosphorylated PLC-γ but not PI3K into membrane localized microclusters

**Figure 5:**
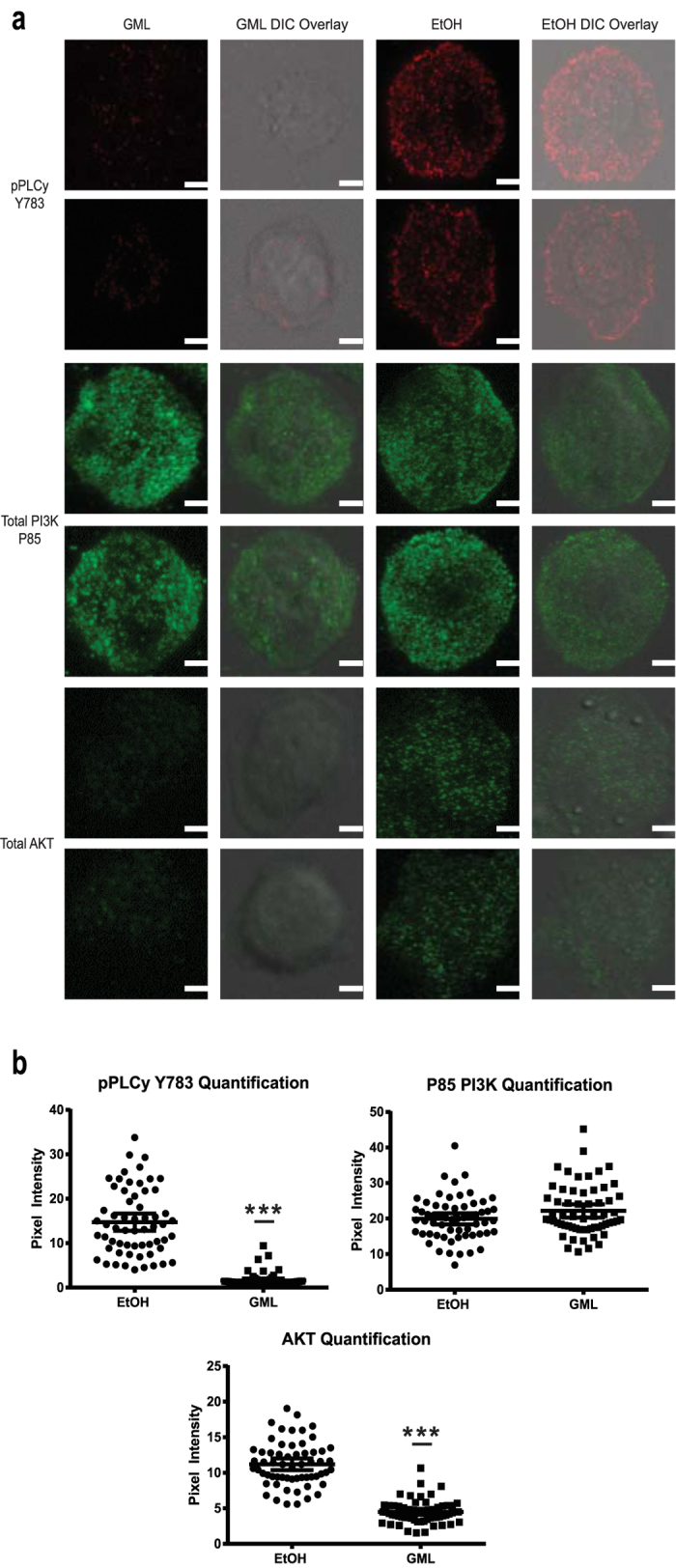
GML prevents membrane clustering of PLC-γ1 and AKT while sparing PI3K. (**a**) APBTs treated with 10 μg/ml of GML or 0.1% ethanol were stimulated in glass covered chamber slides. They were then fixed, permeabilized, and stained with antibody specific for phosphorylated PLC-γ1 Y783, total P85 subunit of PI3K, and total AKT. Cells were imaged using TIRF microscopy. Individual cells are shown. White bar scale indicates 4 μm in length. (**b**) Pixel intensities of images obtained in (**a**) were quantified using ImageJ. Pixel intensities on the median axis of each cell were quantified and averaged. Scatter plot distributions with 95% confidence intervals of 60 cells from 2 independent experiments are shown. *** denotes p < 0.001 in Student’s t test.

PLC-γ1 is highly linked to LAT for its enzymatic activation and is co-localized with LAT at the molecular, nanocluster, and microcluster level upon TCR stimulation^16^,^17^,^21^,^27^,^29^,^30^,^31^,^32^,^33^. Additionally, PI3K and AKT are indirectly linked to these microclusters via association with SLP-76^19^. Hence, we hypothesized that GML mediated suppression of calcium influx and reduced phosphorylation of AKT and p85 subunit of PI3K are due to the inability of PLC-γ1, AKT, and PI3K to form macro scale microclusters similar to our observation with LAT microclusters. To test this, we utilized TIRF microscopy to visualize clustering of these proteins at the cell membrane. GML drastically decreases clustering of phosphorylated PLC-γ1 Y783 and total AKT in both individual cells and pixel quantification of the median cell axis for 60 cells (Fig. 5a,b). In contrast, GML treated cells have similar levels of total p85 subunit of PI3K clustering compared to ethanol control (Fig. 5a,b). Hence, GML inhibits PLC-γ1 enzymatic activity by preventing PLC-γ1 clustering in LAT mediated microclusters (>25 protein molecules) while sparing the protein interaction with small nano scale clusters (<5 protein molecules) that are sufficient to drive protein phosphorylation. While GML does not affect the PI3K clustering at a macro level, the lack of total PI3K phosphorylation and activation results in reduced AKT clustering. Altogether, GML exerts its selective inhibition of TCR induced signaling cascade by preventing LAT and associated protein partners from forming signaling microclusters at the plasma membrane.

### GML disrupts T cell plasma membrane dynamics

Our observation that GML treatment drastically prevents clustering of multiple signaling proteins at the plasma membrane led us to investigate whether GML disrupts plasma membrane lipid organization. GML has been postulated to exert its antimicrobial effects via disruption of pathogen lipid membranes due to its homologous structure to membrane phospholipid^2^. Lipids in the plasma membrane exist on a spectrum ranging between a static ordered state and a dynamic disordered state. To measure how GML alters the distribution of lipid states, we utilized the cell membrane dye, Di-4-ANEPPDHQ, which has different fluorescent properties based on its incorporation into ordered vs. disordered lipid phases^34^. We also used a treatment consisting of 7-ketocholesterol, cholesterol, and methyl-β-cyclodextrin as a positive control because this treatment is known to disrupt ordered lipid domains^35^.

**Figure 6:**
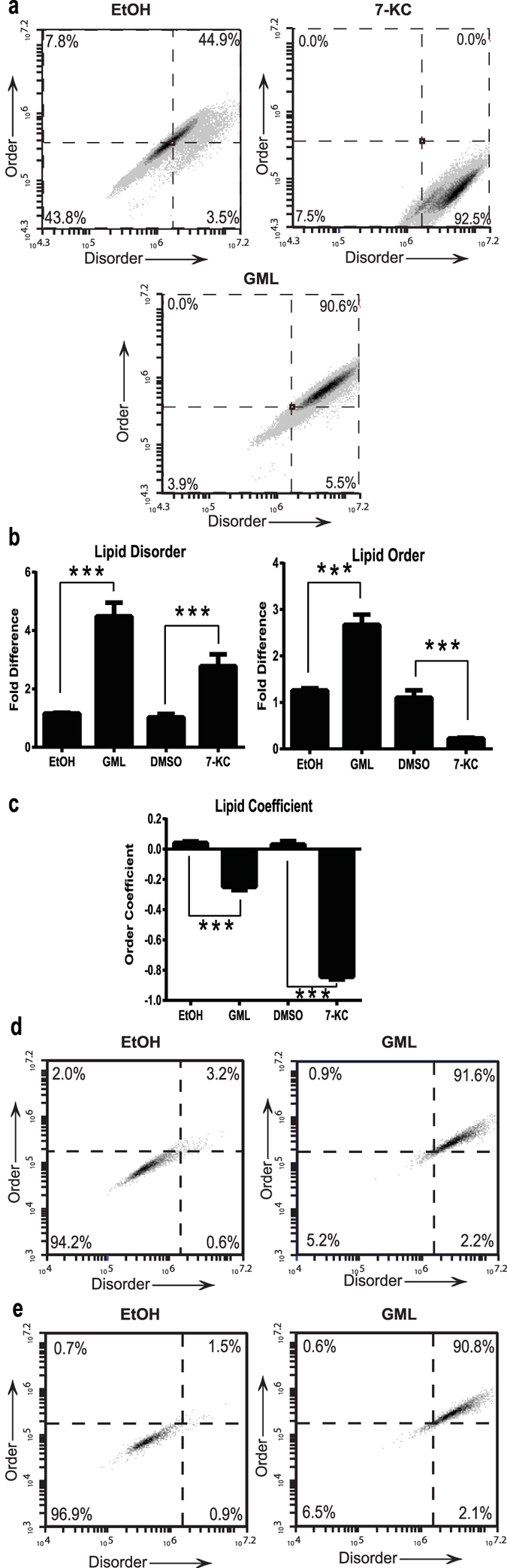
GML disrupts lipid dynamics at the plasma membrane. APBTs treated with 10 μg/ml of GML, 0.1% ethanol, or 7-ketocholesterol were stained with Di-4-ANEPPDHQ. Fluorescent emissions in the green region for ordered lipid domains and red region for disordered lipid domains were measured using flow cytometry. Lipid coefficient that is dependent upon both ordered and disordered measurements. **(a)** shows representative plots with lipid order on the x axis and disorder on the y axis. **(b)** shows lipid coefficient values, and **(c)** shows compiled normalized ordered and disordered measurements for 4 independent experiments. *** denotes p < 0.001 in Student’s t test. **(d,e)** shows lipid order and disorder plots in CD4+ and CD8+ cells respectively.

Interestingly, we observed that GML treated cells have a paradoxical increase in both disordered and ordered lipid domains compared to ethanol control. In contrast, the positive control 7-ketocholesterol treatment resulted in increased disorder and decreased order (Fig. 6a,b). As assessed by the lipid order coefficient, both the 7-ketocholesterol and GML treatment caused overall disorder, but the 7-ketocholesterol treatment had a larger magnitude towards disorder than GML (Fig. 6c). GML induced paradoxical increase in both order and disorder is dose dependent and occurs in both CD4+ and CD8 + T cell populations (Fig. 6d,e, and Supplemental Fig. S3). Additionally, we observed a similar increase of both lipid order and disorder in GML treated cells without TCR stimulation (see Supplemental Fig. S4). Together, these data suggest that GML alters dynamic ordered and disordered lipid regions in the T cell plasma membrane.

## Discussion

GML is an antimicrobial with activity against a variety of pathogens. However, few studies have examined the direct consequence of GML treatment on human immune cell functions. To fill this knowledge gap, we mechanistically examined the effects of GML treatment on human T cell effector functions. We found that GML treatment drastically altered the lipid phase dynamics in the plasma membrane of human T cells. This resulted in a targeted inhibition of microcluster formation and T cell signaling. Ultimately, GML mediated changes in membrane dynamics and cellular signaling translated to deficiencies in cytokine production. Our observations fit with the few studies that demonstrated GML reduces T cell proliferation and production of the second messenger molecule IP_3_^13^,^14^. However, our data greatly expand our mechanistic understanding of how GML alters T cell activation.

The paradoxical increase in both ordered and disordered lipid regions suggest that GML blocks lipids from transitioning between the two states. In the fluid mosaic model, lipid molecules exist on a dynamic equilibrium between the ordered phase with little molecular movement and the disordered phase with dynamic movement^36^. Ordered membrane regions are characterized by highly ordered phase lipids centrally and more disordered phase lipids in the periphery where the ordered lipid regions are in equilibrium with disordered regions^37^. We hypothesize that GML partitions ordered and disordered regions from each other. The disruption of the balance between lipid fluid states by GML causes the paradoxical overall increase in membrane disorder and order.

The disruption of the lipid phase states by GML impacts the formation of LAT nucleated microclusters. LAT is constitutively associated with ordered regions in the membrane^38^,^39^. This interaction between LAT and ordered lipids is necessary to form LAT nanoclusters, which are comprised of less than ten LAT molecules and its direct and indirect ligands such as PLC-γ1 or PI3K. Upon TCR activation, these nanoclusters aggregate into large microcluster units that contain hundreds of LAT molecules^27^. Microcluster formation requires proper lipid interactions however this process is highly complex. Depletion of ordered lipid regions by 7-ketocholesterol expectedly results in decreased LAT microcluster formation due to a reduced association of LAT with ordered lipids in nanocluster units^35^. GML does not affect bulk LAT or PLC-γ1 phosphorylation or LAT phosphorylation at the membrane, suggesting that these nanocluster units are intact. GML potentially disrupts LAT microcluster formation in a much more dynamic process. While GML may inhibit LAT and PLC-γ1 microcluster formation by causing protein dissociation from the plasma membrane due to similar effects seen with other exogenously added lipids^40^,^41^, the paradoxical increase of both ordered and disordered lipid regions by GML lends to the hypothesis that GML prevents aggregation of LAT nanocluster units by preventing the proper lipid-lipid interactions necessary to form microclusters. The dissonance between microcluster imaging and bulk protein phosphorylation indicates that the formation of nanocluster and microcluster units are separable events. This is supported by other reports showing that while T cells deficient in SOS1 have reduced bulk phosphorylated tyrosine residues while having similar microcluster formation of phosphorylated tyrosine proteins compared to wild type^28^. Thus, changes in bulk protein concentration or phosphorylation do not directly correlate with microcluster aggregation but instead reflect nanocluster formation.

T cells lacking GRB2, an adapter protein critical for LAT dimerization, have a signaling defect that is similar to GML treated cells characterized by severely reduced calcium influx and an inhibition of LAT microcluster formation, resulting in a lack of recruitment of PLC-γ1 to the LAT complexes. In contrast to our findings, GRB2 deficient cells have near normal phosphorylation of AKT, and slightly reduced phosphorylation of PLC-γ1 on tyrosine 783^21^. This suggests that inhibition of LAT microclusters by both GRB2 deficiency and GML treatment reduces calcium signaling, resulting in dysfunctional cytokine output. However GRB2 deficiency and GML treatment selectively disrupt different downstream pathways. This difference is likely caused by GRB2 deficiency disturbs both the formation of nanoclusters, which are not visible by TIRF microscopy, in addition to larger aggregated microclusters, whereas GML is nanocluster sparing.

Surprisingly however GML does not have an effect on SLP-76 and PI3K clustering. While we have previously found that PI3K clustering is co-localized with LAT^42^, the lack of inhibition of PI3K microcluster formation in GML treated cells suggest that PI3K and LAT clustering are separable events. Additionally, while the clustering SLP-76 is canonically considered to be dependent upon LAT via its interaction with adapter proteins such as GADS^43^, our data show that SLP-76 cluster independently of LAT. In support of this, the kinetics of TCR induced phosphorylation of SLP-76 is distinct from LAT, suggesting that the two adapter proteins have additional independent properties^31^. Moreover, T cells treated with Latrunculin A, an actin polymerization inhibitor, have impaired LAT clustering but intact SLP-76 clustering^27^. Finally, we have recently published that GADS deficient T cells have severely impaired association of SLP-76 with LAT but have normal SLP-76 phosphorylation and downstream actin polymerization, suggesting that SLP-76 without LAT is still able to associate with its signaling partners and drive WASp activation^44^. Finally, A possible explanation is that SLP-76 interacts with the cytoplasmic tail of the cell surface molecule CD6 to cluster in the absence of LAT^45^. In all, the differential formation of microclusters of various signaling proteins cause the targeted PLC-γ1 and PI3K-AKT signaling defects in GML treated cells.

Collectively, we found that GML is a potent suppressor of T cell functions and signaling by altering T cell plasma membrane lipid dynamics. These findings establish GML as a novel immunosuppressant, in addition to being an antimicrobial agent. Understanding the direct impact of this chemical on human health is integral for the proper utilization of GML in its various roles. GML comes in regular contact with the public either topically or by oral ingestion due to its ubiquitous uses in cosmetic, food, and homeopathic products. In human breast milk, GML is generated from triglycerides with 12 carbon fatty acid side chains by lipases in the gastrointestinal tract at doses between 1–2 mg/ml, which is much higher than concentrations used in our studies^46^,^47^. Formula fed infants will have higher *in vivo* concentrations of GML, since the major source of fatty acids in nearly all infant formula is coconut oil, which is primarily composed of 12 carbon chain fatty acid triglycerides. Additionally, GML is administered at 50 mg/mL in the studies examining SIV infection^6^,^10^ and approximately 8 mg of GML are used during vaginal application with tampons^11^. GML may have unintended anti-inflammatory functions in these formulations. Finally, topical GML is a possible therapeutic for autoimmune and type IV hypersensitivity skin disorders such as psoriasis, poison ivy sensitivity and latex allergies, while oral GML has the potential to ameliorate excessive gastrointestinal inflammation in diseases such as Crohn’s disease or ulcerative colitis In conclusion, we demonstrate that GML treatment potently suppresses T cell functions and signaling by disturbing lipid dynamics in the plasma membrane suggesting that GML may be a potent immunosuppressant for therapeutic and commercial applications.

## Materials and Methods

### Primary human T cell isolation, GML preparation and viability assay

PBMCs were isolated from the whole blood of healthy donors that have consented for blood donation at the DeGowin Blood Center at the University of Iowa Hospitals and Clinics. Donors are anonymous and have provided written informed consent to allow their cells that are normally discarded to be used in research studies. The recruitment protocol and written informed consent document were approved by the Institutional Review Board for the University of Iowa. All samples were provided to investigators de-identified. Therefore, further IRB approval for the use of the cells by the investigators was not needed based on Federal Regulation 46.101.B4. Hence, all experiments were performed in accordance with approved guidelines. PBMCs were isolated using Hypaque-Ficoll density-gradient separation. T cells were then expanded and activated using anti-CD3 and anti-CD28 coated beads (Invitrogen) and human IL-2 for five days. Cells were then re-suspended in fresh media without stimulatory beads and IL-2 for 24 hours and are termed activated peripheral blood T cells (APBTs). APBTs were resuspended in serum free media before GML treatment. GML was solubilized at room temperature in 95% ethanol and diluted into the appropriate working concentration. 95% ethanol was added as comparative vehicle control at final concentrations that did not exceed 0.5%. APBTs were treated with ethanol or various doses of GML and plated for 24 hours. Cellular viability was measured by Tryphan blue exclusion.

### Enzyme-linked immuno assay (ELISA) detection of cytokines

0.5% or 0.2% ethanol control or various doses of GML were added to APBTs in serum free media. The cells were then stimulated with plate-bound anti-CD3 and soluble anti-CD28 (Biolegend) for 24 hours. Both anti-CD3 and anti-CD28 antibodies were used because APBT cytokine production is optimally detectable using both anti-CD3 and anti-CD28 antibodies and not anti-CD3 alone^48^. Protein concentrations of IL-2, IFN-γ, IL-10, and TNF-α in the media supernatants were measured by standard TMB ELISA. Due to high variability in cytokine production from independent human donors, cytokine levels were normalized to cytokine production in ethanol control groups.

### Immunoblotting

APBTs were treated with 10 μg/ml of GML or 0.1% ethanol control and then incubated on ice with anti-CD3 and anti-CD4 for 30 minutes. Cells were then warmed at 37 °C for 10 minutes and stimulated using IgG crosslinking antibody for various times. Both anti-CD3 and anti-CD4 antibodies were used because detectable TCR-mediated signaling is optimally detectable using both CD3 and CD4 antibodies and not CD3 alone^31^. Cells were then lysed with 2X sample buffer, heated to 95 °C, and sonicated. Cell lysates were separated by polyacrylamide gel electrophoresis and transferred to PVDF. The membranes were blocked in 0.5X SEA BLOCK blocking buffer diluted in PBS. Primary antibodies were incubated overnight at 4 °C. Secondary antibodies incubated with the membrane for 30 minutes at room temperature and then imaged using Licor Odyssey (Lincoln, NE, USA). At least five independent replicates with different human donors were performed for each experiment. Immunoblot band intensity was quantified using Odyssey’s v3.0 software and phosphorylated form of proteins was normalized to GAPDH. Normalization of phospho-specific proteins to pan forms may give misleading results due to the inability of pan antibodies to detect phosphorylated forms and the steric hindrance that can occur when two antibodies bind to the same protein. To minimize variability between human donors, band intensities were further normalized to maximal band intensity of ethanol vehicle control. The following antibodies were used for immunoblotting: SLP-76 pY128 (clone J141-668.36.58, BD Pharmingen), AKT pThr 308 (Cell Signaling), AKT pS473 (Invitrogen), ZAP-70 pY493 (Cell Signaling), LAT pY191 (Millipore), PI3K P85 pY458/P55 pY199 (Cell Signaling), PLC-γ pY783 (Cell Signaling), P38 MAPK pT180/Y182 (Cell Signaling), Erk 1/2 pT185/Y187 (Invitrogen), and GAPDH (Meridian Life Sciences). IRDye 800CW or IRDye680-conjugated secondary antibodies were used (Licor). Phospho-specific LAT pY191 antibody was used to measure LAT phosphorylation levels due to its robust phosphorylation kinetics upon TCR activation^48^.

### Calcium Influx

Calcium influx assays were performed as previously described^21^. In short, APBTs were incubated Fluo-4 M calcium dye (Thermo Fisher) (5 μM) and probenecid (2.5 mM) at 37 °C for 45 minutes. Cells were washed and anti-CD3 (2 μg/ml), anti-CD4 (2 μg/ml), 2.5 mM probenecid, and various doses of GML or 0.1% ethanol control were added. Cells were incubated on ice for 15 minutes and then warmed at 37 °C for 5 minutes before using an Accuri C6 flow cytometer to detect Fluo-4M fluorescence in the FL1 channel. Basal cytoplasmic calcium levels were measured for 35 seconds, and then cells were stimulated with the addition of IgG crosslinking antibody for 6 minutes to detect induction of calcium dye fluorescence. Ionomycin was then added to control for total cellular Fluo-4M levels. Mean fluorescent intensity values each experiment group were averaged and graphed from three independent experiments from three different human donors.

### Total Internal reflection fluorescence (TIRF) microscopy

APBTs were stimulated in the presence of 0.1% ethanol control or 10 μg/ml GML with plate-bound anti-CD3 on glass coverslips, fixed with 4% paraformaldehyde, permeabilized with 0.25% Triton-X, and stained as described below. To detect microcluster formation of TCR signaling proteins, antibodies specific for LAT pY226 (BD Pharmingen), PLC-γ pY783 (Cell Signaling, total P85 PI3K (Millipore), and total AKT (Cell signaling) were incubated with fixed and permeabilized cells overnight for 4 °C. Subsequently, conjugated secondary antibodies DyLight 488 Goat anti-rabbit IgG and Alexa Fluor 568 goat anti-mouse IgG1 (Thermo Fisher) were incubated with the cells at room temperature for 2 hours. All images were captured by the Leica AM TIRF MC system using 100x magnification oil immersion objective lens at room temperature at the University of Iowa Central Microscopy Research Facility. Phospho-specific LAT pY226 antibody was used due to its ability to robustly image LAT micrcoclusters in TIRF^17^.

### Image quantification

TIRF microscopy images were processed and analyzed using ImageJ. Quantification of membrane clustering was done by measuring mean pixel intensity in the longest axis of cells for 60 cells total from at least 3 independent experiments. Similar quantification methods have been used in other published reports^17^,^21^,^49^.

### Membrane order vs. disorder measurement

APBTs were washed and resuspended in RPMI without phenol red. Cells were treated with 10 μg/ml GML, 0.1% ethanol vehicle control, or 7-ketocholesterol-cholesterol (7KC) mixture positive control at 37 °C for 30 minutes. The 7-ketocholesterol-cholesterol mixture was prepared as previously described^50^. Briefly, 15 mg/ml of 7KC and cholesterol (Avanti Lipids) were mixed at a 2:1 ratio at room temperature for 30 minutes, then added to heated 50 mg/ml methyl-β-cyclodextrin (Sigma Aldrich) at 80 °C in PBS for a sterol concentration of 1.5 mg/ml. 15ul of this mixture was added to 1 ml of APBT suspension for a final sterol concentration of 22.5 μg/ml and final methyl-β-cyclodextrin concentration of 0.5 mM. This setup with the stated sterol and methyl-β-cyclodextrin concentrations is effective at targeting 7KC to the membrane without depletion of endogenous membrane cholesterol^35^. After treatment, APBTs were stained with 1 μM of Di-4-ANEPPDHQ at 37 °C for 1 hour. Di-4-ANEPPDHQ is a membrane intercalating dye that has an emission peak at ~490 nm when it is incorporated in ordered phase membrane domains and ~690 nm if in disordered membrane domains^51^. The cells were then washed, warmed at 37 °C, and stimulated using anti-CD3, anti-CD4, and IgG crosslinking antibodies. The Accuri C6 Flow Cytometer was then used to measure mean fluorescence intensities (MFI) for 10,000 events in FL1 channel and FL3 channel for ordered and disordered lipid domains respectively. Flow plots were plotted with disorder in the x axis and order in the y axis to depict shifts in cell populations. MFI from 4 independent experiments were normalized to no treatment internal controls and then averaged for statistical analysis. To incorporate both ordered and disordered measurements into a single value for quantification, the following formula for ordered coefficient was used:


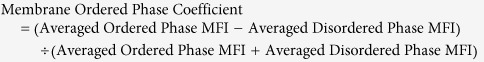


Measurements of lipid order and disorder in CD4+ and CD8+ T cells specifically were done as previously described^51^. Briefly, cells were stained with Di-4-ANEPPDHQ and anti-CD4 or CD8 with Alexa 647 fluorescent secondary ab. CD4/CD8 positive cells were selected on forward scatter and FL-4 fluorescence signal in flow cytometry after appropriate compensation. Cells stained with only Di-4-ANEPPDHQ dye without anti-CD4/CD8 antibodies served as negative control. Cell gating is shown in Supplemental Figure 3B.

### Statistical Analysis

Student’s t test was done in Microsoft excel by use of two-tailed test assuming equal variance. One way ANOVA tests and 95% confidence interval was done in Graphpad Prism.

### Ethics Statement

All experimental protocols pertaining to tissues isolated from human samples are done in accordance with approved guidelines. All experimental protocols were approved by the Institutional Review Board for the University of Iowa. Written informed consent was obtained from all blood donor subjects.

## Additional Information

**How to cite this article**: Zhang, M. S. *et al.* Glycerol Monolaurate (GML) inhibits human T cell signaling and function by disrupting lipid dynamics. *Sci. Rep.* **6**, 30225; doi: 10.1038/srep30225 (2016).

## Supplementary Material

Supplementary Information
