# Supplementary material for: Glycerol Monolaurate (GML) inhibits human T cell signaling and function by disrupting lipid dynamics
Source: Sci Rep. 2016 Jul 26;6:30225. doi: 10.1038/srep30225 (PMC4960522; doi:10.1038/srep30225)
Supplement: Supplementary Information [file srep30225-s1.pdf]

Supplementary Data for Glycerol Monolaurate (GML) inhibits human T cell signaling and function by disrupting lipid dynamics

Michael S. Zhang[1], Aline Sandouk[1], Jon C.D. Houtman[1,]*

[1] Department of Microbiology, Carver College of Medicine, University of Iowa, Iowa City, Iowa 52242, United States.[‡]

To whom correspondence should be addressed: Jon Houtman

University of Iowa

Carver College of Medicine

2210 MERF

Iowa City, IA 52242

Phone: (319) 335-7780

Email: Jon-houtman@uiowa.edu

Supplemental Figure 1

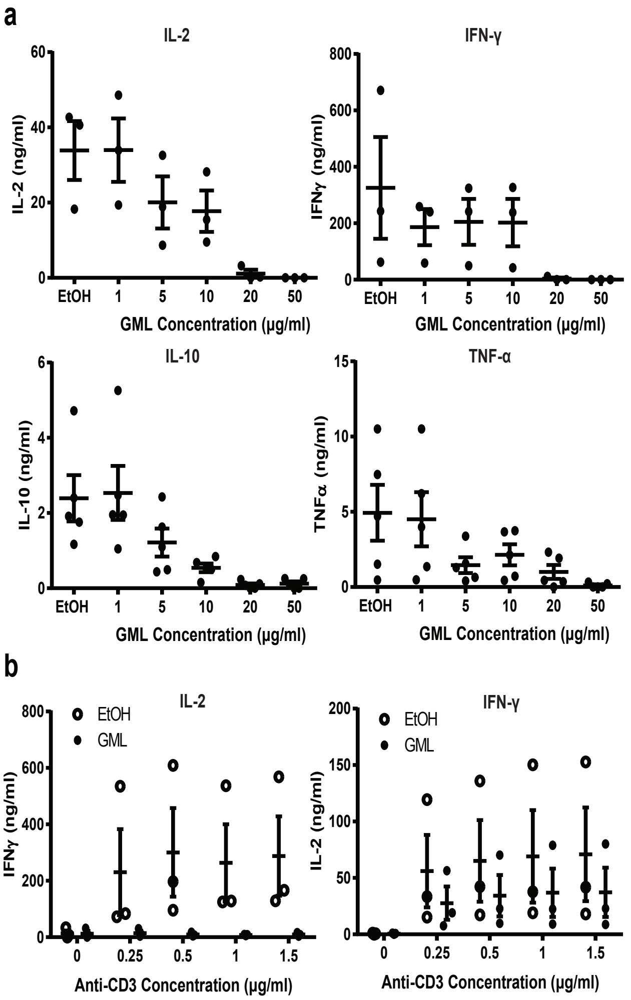

Fig S1: Distribution of raw values of GML mediated suppression of cytokine production. **(A)**. APBTs were treated with GML or ethanol vehicle control and stimulated as described Fig 1A. Distribution of cytokine concentrations from at least 3 individual human donors is shown: IL-2 (Upper Left), IFN-γ (Upper Right), IL-10 (Lower Left), and TNF-α (Lower Right). **(B)**. APBTs were treated with treated with GML or ethanol vehicle control and stimulated as described Fig 1B. Distribution of Distribution of cytokine concentrations from at least 3 individual human donors is shown: IL-2 (Left) and IFN-γ (Right).

Supplemental Figure 2

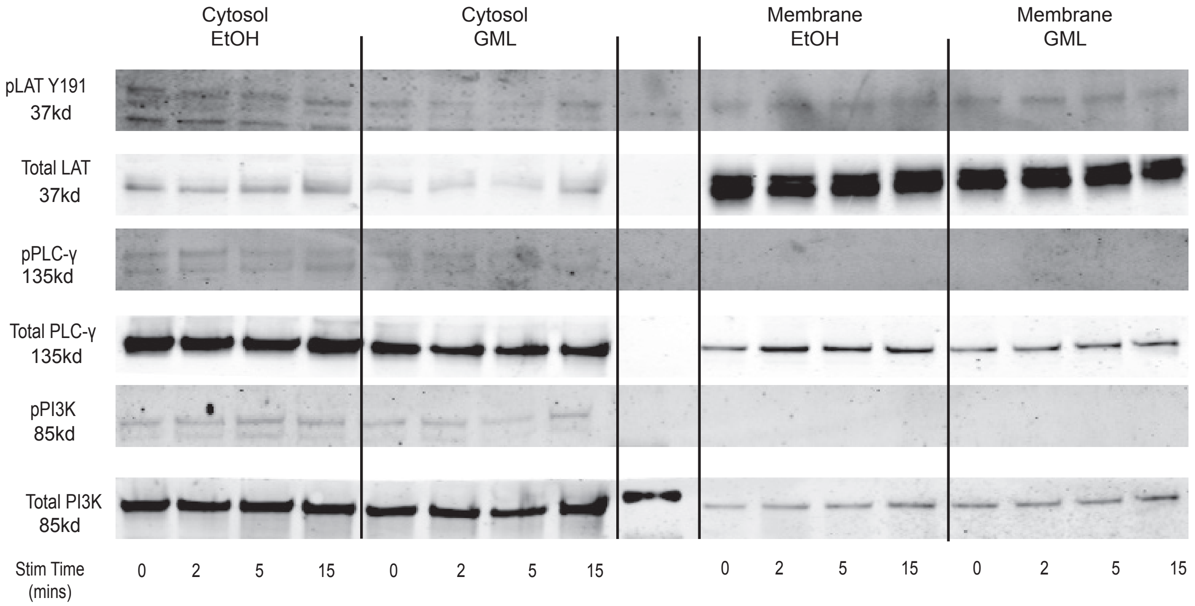

Fig S2: GML does not alter LAT phosphorylation at the membrane. APBTs treated with 10μg/ml of GML or 0.1% ethanol and thenn stimulated by crosslinking anti-CD3 (2 μg/ml) and anti-CD4 (2 μg/ml) with IgG for 0, 2, 5, and 15 minutes. Cell fractionation was done using Qproteome Cell Compartment Kit (Qiagen) according to manufacturer's instructions. Representative immunoblots from 3 independent experiments utilizing different human donors are shown. Cytosolic fractions are on the left and the membrane fractions are on the right with the loading ladder inbetween.

Supplemental Figure 3

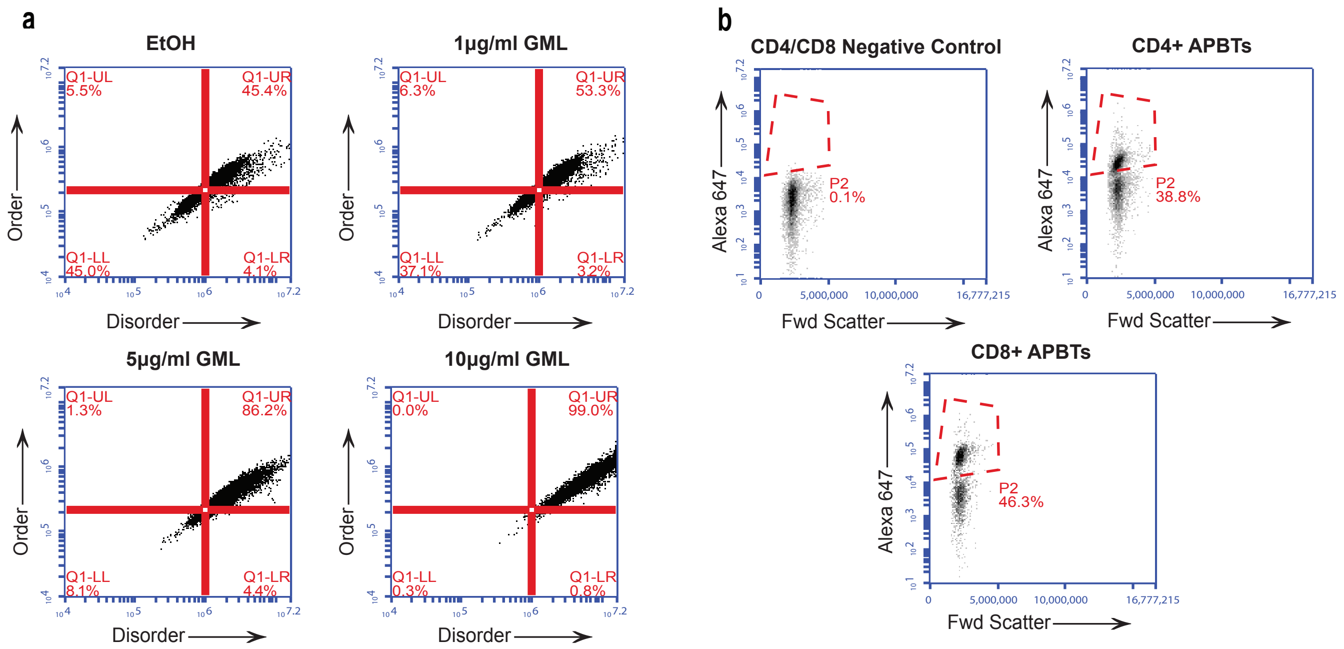

Fig S3: GML's effects on lipid dynamics is dose dependent and occurs in both CD4 and CD8 T cells. **(A)**. APBTs treated with 1, 5, or 10μg/ml of GML, or 0.1% ethanol were stained with Di-4-ANEPPDHQ. They were then stimulated by crosslinking anti-CD3 (2 μg/ml) and anti-CD4 (2 μg/ml) with IgG. Fluorescent emissions in the green region for ordered lipid domains and red region for disordered lipid domains were measured using flow cytometry. Representative plots with lipid order on the x axis and disorder on the y axis are shown. **(B)** shows the gating strategy to isolate CD4 or CD8+ APBTs. Cells were stained with Di-4-ANEPPDHQ and anti-CD4 or CD8 with Alexa 647 fluorescent secondary ab. CD4/CD8 positive cells were selected on forward scatter and FL-4 fluorescence signal in flow cytometry after appropriate compensation. Cells stained with only Di-4-ANEPPDHQ dye without anti-CD4/CD8 antibodies served as negative control.

Supplemental Figure 4

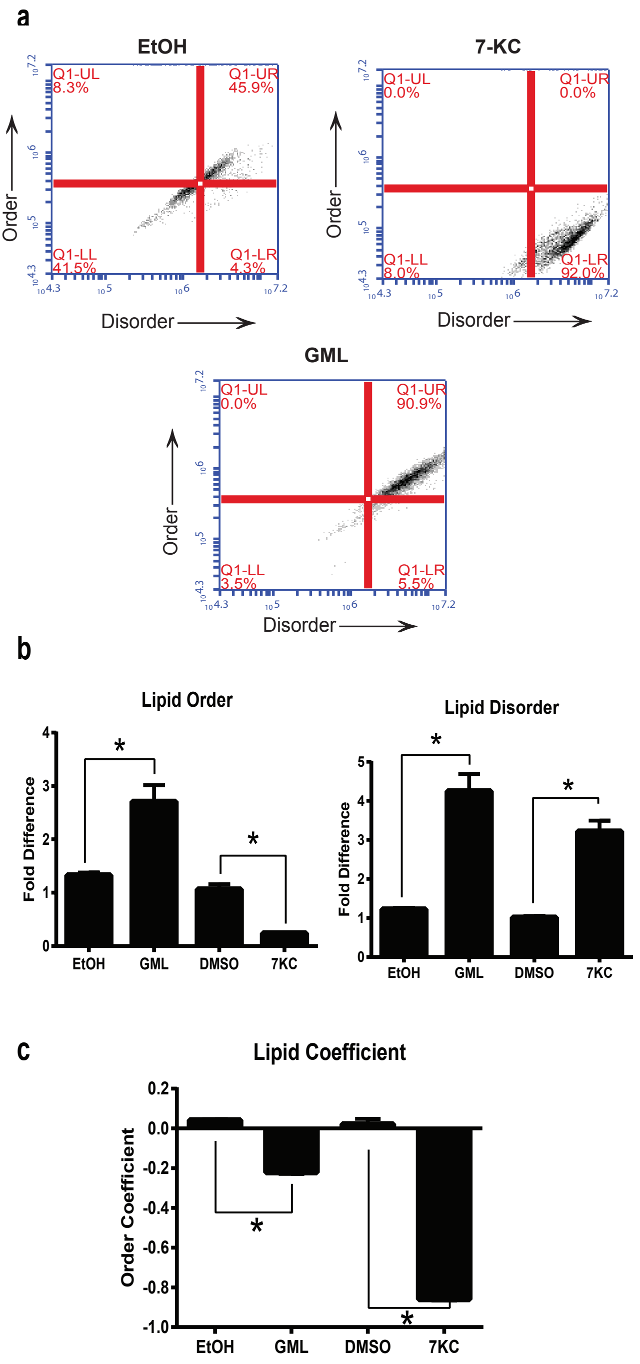

Fig S4: GML disrupts plasma membrane lipid dynamics in resting cells without restimulation. APBTs were treated with GML, ethanol, or 7-ketocholesterol, stained with the membrane dye Di-4-ANEPPDHQ, and underwent measurements for lipid order and disorder as described in Figure 7. **(A)**. shows representative histograms, **(B)** shows compiled normalized measurements from 4 independent experiments, and **(C)** shows lipid coefficient values. * denotes p<0.05 in Student t's test.
